# Effective Pain Management in Polyarteritis Nodosa (PAN) Utilizing Lumbar Sympathetic Blocks: A Case Report

**DOI:** 10.5812/aapm-144910

**Published:** 2025-04-12

**Authors:** Masood Mohseni, Behnaz Karimi, Farnad Imani

**Affiliations:** 1 Pain Research Center, Department of Anesthesiology and Pain Medicine, School of Medicine, Iran University of Medical Sciences, Tehran, Iran

**Keywords:** Chronic Pain, Vasculitis, Pain, Sympathectomy, Vasculitis, Sympathetic Block, Polyarteritis Nodosa

## Abstract

**Introduction:**

We presented a 39-year-old man with polyarteritis nodosa (PAN) experienced significant leg pain unresponsive to oral medications.

**Case Presentation:**

Two sequential lumbar sympathetic blocks with ropivacaine and triamcinolone resulted in over 70% pain reduction (NRS 9-10 to NRS 3) over a three-month follow-up period.

**Conclusions:**

To our knowledge, this is the first report showing the efficacy of lumbar sympathetic block in controlling PAN-associated pain.

## 1. Introduction

Polyarteritis nodosa (PAN) is a rare systemic necrotizing vasculitis affecting medium-sized arteries, resulting in multifaceted clinical manifestations. The etiology of pain in PAN is multifactorial, stemming from vascular inflammation and subsequent ischemia affecting various organs and tissues (1). Patients commonly experience musculoskeletal pain, neuropathic pain, and abdominal pain, which can be severe and refractory to standard analgesic regimens.

Traditional pain management strategies in PAN primarily involve the use of high-dose corticosteroids and immunosuppressive agents to control the underlying inflammatory process (2). Additionally, non-steroidal anti-inflammatory drugs (NSAIDs), opioids, and adjunctive therapies are employed to alleviate pain. However, these treatments often offer limited relief, and their long-term use may lead to significant side effects and complications.

The intricate nature of pain in PAN necessitates a comprehensive and multidisciplinary approach to address the diverse aspects of pain experienced by affected individuals. Given the complexity of the pain mechanisms involved in PAN, alternative and innovative approaches are continuously being explored to improve pain control and enhance patients' overall well-being (3). Mechanistically, lumbar sympathetic block may disrupt aberrant pain signaling and modulate vascular inflammation, improving blood flow and mitigating ischemic pain (4).

This paper presents a patient diagnosed with PAN who presented with persistent leg pain unresponsive to oral analgesics and daily methylprednisolone, and who responded satisfactorily to two sequential lumbar sympathetic blocks, spaced two weeks apart.

## 2. Case Presentation

A 39-year-old healthy man with no prior medical history developed tender subcutaneous nodules, mostly affecting both legs and the back area, in August 2022. The nodules were predominantly present on the back of the thigh and calf, but they also spread across the dorsal and plantar surfaces of the feet, heels, legs, and thighs.

The patient complained of fatigue, loss of appetite, weight loss, and generalized weakness. During the illness, his right eyesight significantly diminished and remains impaired to this day.

**Figure 1.**
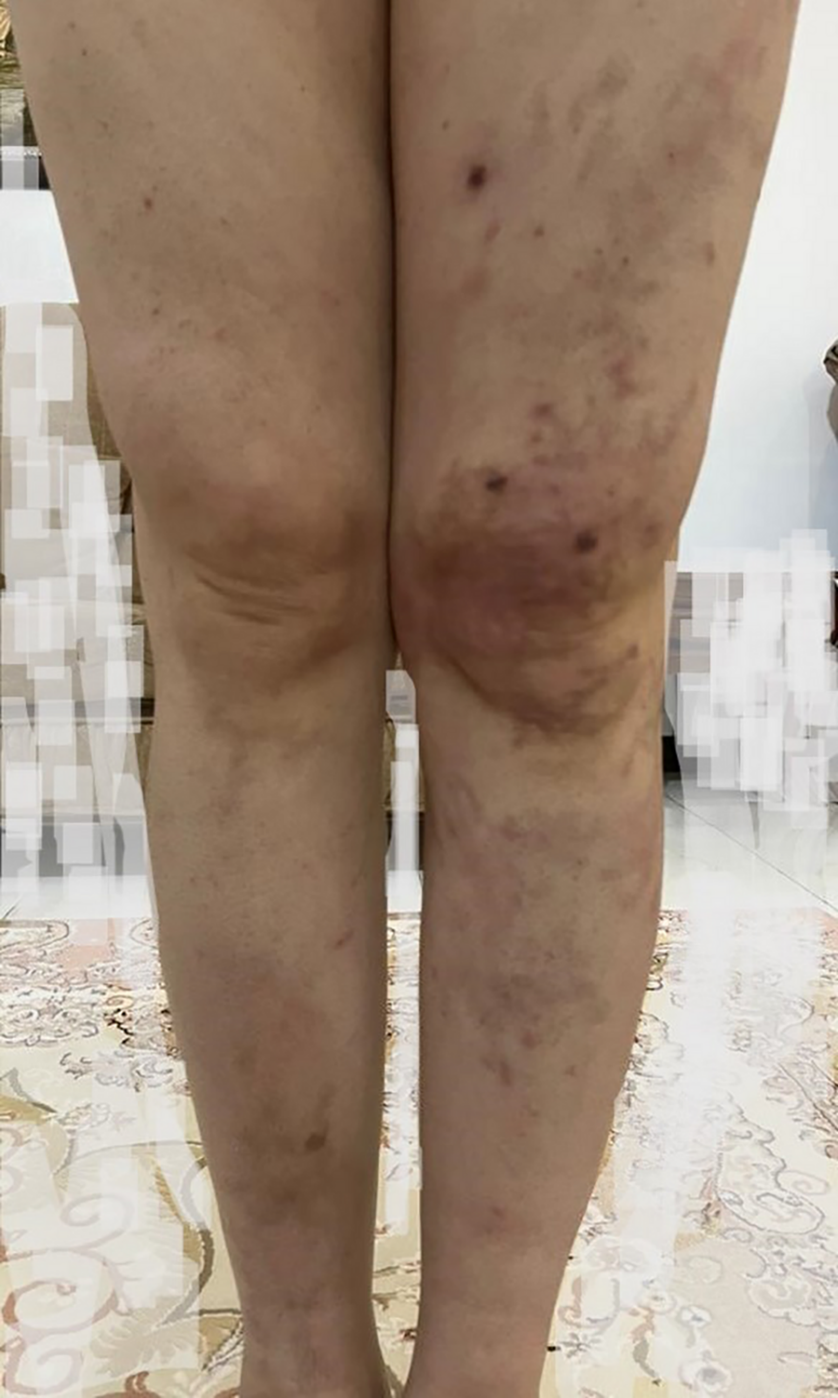
Cutaneous nodules spreading on both lower limbs.

He experienced severe, heavy, aching pain in the affected areas, mainly on the left side, which immobilized him in the morning (rated at NRS 9 - 10). He had to take analgesics while in bed, and after approximately two hours, when the analgesics took effect, he could walk on his feet. He also felt uncomfortable paresthesia and tingling in his toes. In his physical examination, we found no abnormality except for cutaneous nodules spreading on the trunk and both lower limbs, mostly on the calves and feet (Figure 1). Sensory and motor examinations, as well as reflexes, were normal.

After about six months from symptom initiation, he received a diagnosis of PAN. Treatment included prednisolone 50 mg, gabapentin 300 mg, diclofenac 300 to 500 mg, and daily calcium-D (500 mg/200 IU). The patient received intravenous Zytux 500 mg weekly for four episodes, which reduced his pain by 10%. Subsequently, he was referred to our pain clinic and underwent two episodes of unilateral lumbar sympathetic block, two weeks apart, with 15 cc of 0.2% ropivacaine and 80 mg of triamcinolone for each injection. The pain responded dramatically to the block, and in follow-up visits, the patient reported more than 70% pain reduction with an NRS equal to 3.

### 2.1. Procedure of Lumbar Sympathetic Block

The patient was placed in the prone position on the fluoroscopy table. Standard monitors were applied, including pulse oximetry, blood pressure cuff, and ECG leads. The lumbar area was cleaned with antiseptic solution and draped in a sterile fashion. Local anesthesia (1% lidocaine) was infiltrated into the skin and subcutaneous tissue at the planned needle insertion site to ensure patient comfort during the procedure.

Using fluoroscopy, the L3 vertebral body was identified on the left side, and the correct anatomical landmarks were confirmed. A 22-gauge, 15-cm Chiba needle was advanced under fluoroscopic guidance toward the anterolateral aspect of the L3 vertebral body, aiming for the region of the lumbar sympathetic chain. Once the needle tip was appropriately positioned, a small amount of non-ionic contrast dye was injected to confirm correct needle placement and ensure there was no intravascular or intrathecal uptake. After confirming satisfactory contrast spread, 15 mL of a solution containing 0.2% ropivacaine and 80 mg of triamcinolone was injected slowly through the needle. The needle was carefully removed, and a sterile dressing was applied to the injection site. The patient tolerated the procedure well without any complications.

Currently, three months have passed, and the 70% pain relief persists. His analgesics have been tapered, and he is receiving a daily regimen of prednisolone 5 mg, gabapentin 300 mg, mycophenolate 500 mg, and Dino (a supplement to protect the immune and nervous system).

## 3. Discussion

The case's favorable response to lumbar sympathetic blocks presents a potential interventional strategy for managing refractory PAN-associated pain. Comparative analysis with conventional treatments highlights the limitations of traditional approaches, where oral medications sometimes fall short in achieving optimal pain control (5).

A lumbar sympathetic block may improve pain management in PAN through several potential mechanisms. The PAN involves necrotizing inflammation of medium-sized arteries, leading to ischemia and subsequent pain. The sympathetic nervous system plays a crucial role in regulating blood flow and pain transmission. By interrupting sympathetic nerve transmission through the lumbar sympathetic block, the procedure can potentially alleviate pain in PAN (6, 7).

This block involves the injection of local anesthetic agents, such as ropivacaine, and corticosteroids like triamcinolone, targeting the sympathetic chain in the lumbar region. By blocking sympathetic nerve impulses, the procedure may disrupt aberrant pain signaling pathways, decrease vasospasm, reduce inflammatory responses, and improve blood flow to the affected areas, consequently mitigating the ischemic pain associated with PAN (8). Additionally, the corticosteroid component can provide anti-inflammatory effects, potentially modulating the underlying vascular inflammation contributing to pain. This pain-relieving effect has been specifically reported in those PAN patients with calf pain (9). This collective action might contribute to the observed pain relief and improved functional outcomes seen in our patient with PAN undergoing lumbar sympathetic blocks.

Further investigation into the mechanisms underlying sympathetic block effectiveness in PAN-associated pain is warranted. Exploring larger cohorts and long-term follow-ups could elucidate the role of these interventions in comprehensive PAN management.

### 3.1. Conclusions

This case report showcases a remarkable (> 70%) reduction in pain severity observed in a PAN patient following lumbar sympathetic blocks. The findings suggest a promising avenue for managing PAN-associated pain refractory to conventional therapy, urging further research to establish its broader applicability.

## Data Availability

The dataset presented in the study is available on request from the corresponding author during submission or after publication. The data are not publicly available due to patient's confidentiality.
